# The Battleship technique: A reliable method to quantify intraarticular distance maps patterns and correlate hindfoot alignment

**DOI:** 10.1002/jeo2.70190

**Published:** 2025-03-07

**Authors:** Ben Efrima, Agustin Barbero, Amit Benady, Yair Green Halimi, Jari Dahmen, Gino M. M. J. Kerkhoffs, Jon Karlsson, Cristian Indino, Camila Maccario, Federico G. Usuelli

**Affiliations:** ^1^ Sackler School of Medicine Tel Aviv University Tel Aviv Israel; ^2^ Division of Orthopaedic Surgery Tel Aviv Ichilov Sourasky Medical Center Tel Aviv Israel; ^3^ Foot and Ankle Department Humanitas San Pio X Hospital Milan Italy; ^4^ Department of Orthopedic Surgery and Sports Medicine, Amsterdam Movement Sciences, Amsterdam UMC, Location AMC University of Amsterdam Amsterdam the Netherlands; ^5^ Academic Center for Evidence‐based Sports Medicine (ACES) Amsterdam UMC Amsterdam the Netherlands; ^6^ Amsterdam Collaboration for Health & Safety in Sports (ACHSS), International Olympic Committee (IOC) Research Center Amsterdam UMC Amsterdam the Netherlands; ^7^ Department of Orthopaedics, Sahlgrenska Academy Gothenburg University Gothenburg Sweden; ^8^ Department for Orthopaedics, Sahlgrenska University Hospital Institute of Clinical Sciences, Sahlgrenska Academy Gothenburg University Gothenburg Sweden

**Keywords:** ankle joint, Battleship technique, distance mapping, osteoarthritis, weight‐bearing computed tomography

## Abstract

**Purpose:**

Weight‐bearing computed tomography (WBCT) creates colour‐coded distance maps (DM) to analyze intraarticular contact areas, offering a detailed assessment of joint surface interactions. However, clinical applications of DM remain underexplored. This study introduces the ‘Battleship technique (BST)’ to evaluate contact area patterns in patients with osteoarthritis (OA) of the talar dome, producing a single point representing the distance map weighted sum (DMWS). The DMWS serves as a potential reference for assessing hindfoot deformities and guiding clinical decisions, including surgical planning and alignment correction. We hypothesize that the BST is reliable for calculating the DMWS and that the DMWS correlates with hindfoot alignment, providing a novel tool to improve the evaluation of complex deformities. The primary aim was to evaluate the reliability of the BST, and the secondary aim was to determine whether the DMWS is influenced by hindfoot alignment.

**Methods:**

Two raters independently calculated DMWS using BST for forty ankle OA patients. Based on DMWS location relative to the joint centre, patients were categorized into coronal (varus/valgus) and sagittal (anterior/posterior) groups. Hindfoot alignment was statistically compared between groups.

**Results:**

Excellent interobserver and intraobserver agreement was observed. Significant differences were found in *α* angle, tibiotalar surface angle (TSA), hindfoot alignment angle (HFA) and talar tilt (TT) (*p* = 0.047, *p* < 0.001, *p* = 0.003 and *p* = 0.04) between coronal groups, and in *β* angle and tibiotalar ratio (TTR) (*p* < 0.001) between sagittal groups. Correlations were identified between DMWS and TSA (*r* = 0.6, *p* < 0.001), TT (*r* = −0.6, *p* < 0.001), *β* angle (*r* = 0.2, *p* < 0.001) and TTR (*r* = −0.4, *p* < 0.001).

**Conclusion:**

The BST reliably calculates the DMWS, correlating with foot and ankle alignment. BST provides a standardized, non‐invasive method to evaluate intraarticular contact patterns, offering valuable insights for preoperative planning and post‐operative assessment. Its integration into practice may enhance surgical precision in complex realignment procedures.

**Level of Evidence:**

Level IV.

**Abbreviations**
3D: three‐dimensional
AL: anterolateral
AM: anteromedial
BST: Battleship technique
COP: centre of pressure
DM: distance mapping
DMC: distance mapping coefficient
DMWS: distance map weighted sum
FEA: finite element analysis
HFA: hindfoot alignment angle
ICC: interclass correlation coefficient
OA: osteoarthritis
PL: posterolateral
PM: posteromedial
SD: standard deviation
TSA: tibiotalar surface angle
TT: talar tilt
TTR: tibiotalar ratio
TWSx: total weighted sum X
TWSy: total weighted sum Y
WBCT: weight‐bearing computed tomography
WSQx: weighted sum for each square along the *X*‐axis
WSQy: weighted sum for each square along the *Y*‐axis

## INTRODUCTION

The tibiotalar joint has a limited capacity to tolerate malalignment and joint incongruency [18]. Consequently, most foot and ankle surgeries focus on restoring the natural, balanced alignment of the ankle and hindfoot. Therefore, meticulous preoperative planning is essential to assess both intra‐articular and extra‐articular deformities. Currently, most surgeons rely on standard weight‐bearing radiographs for preoperative planning. However, this approach is limited by rotational bias and its restricted ability to accurately display complex intra‐articular three‐dimensional (3D) deformities [5, 10, 16]

The introduction of advanced technologies has improved our ability to examine the relationships between malalignment, incongruency, and intra‐articular contact [12]. Weight‐bearing computed tomography (WBCT) provides 3D visualization of joint alignment under load, enabling detailed assessment of intra‐articular deformities across all planes. To enhance the clinical utility of WBCT for deformity analysis and surgical planning, defining a single representative intraarticular centre point of the deformity across the coronal, sagittal and axial planes is crucial. This point could standardize assessments, guide surgical interventions, and help monitor post‐operative outcomes, offering a practical link between advanced imaging and clinical decision‐making.

The integration of WBCT with advanced image analysis software has facilitated the development of distance mapping (DM) [9, 11], an algorithm that quantifies the gap between articulating surfaces. This algorithm employs a colour‐coded system to visually represent the intraarticular contact area, creating a map highlighting variations in contact points within the joint. This technique offers a more detailed understanding of how joint surfaces interact, similar to how a geographical map details terrain variation. Bernasconi et al. and Lintz et al. [2, 15] have applied DM to investigate planovalgus and cavovarus alignment, respectively. They segmented the joint surface into quadrants and discovered that specific areas consistently exhibited reduced gaps between them. This observation suggests that feet with particular alignments display predictable contact patterns between joint contact areas.

Expanding on the utility of DM, Corazza et al. [3] explored how intraarticular distance changes with passive ankle flexion. They compared DM to intraarticular pressure maps and found similar patterns, highlighting DM's potential as a non‐invasive technique for analyzing joint pressure. Similarly, Peiffer et al. [16] conducted a study using 3D finite element analysis (FEA) alongside WBCT and found a correlation between tibio talar malalignment, intraarticular pressure maps and contact maps patterns. This revelation has sparked increased interest among researchers in further exploring DM's capabilities [7, 9, 11, 15]. Despite the promise shown by DM, its clinical applications in day‐to‐day practice still need to be clarified. Addressing this gap requires a refined methodology for analyzing DM patterns to determine whether DM patterns correlate with overall foot alignment. Advancements in this area could improve our comprehension of foot biomechanics, paving the way for innovative diagnostic and therapeutic strategies for foot and ankle conditions.

In the current study, the “Battleship technique” (BST), was employed to analyze DM patterns in patients with OA of the talar dome and provide a single point that represents the DM‐weighted sum. The primary aim of this study was to evaluate the reliability of the BST; the secondary aim was to assess whether the DM‐weighted sum correlates with foot and ankle alignment. It is hypothesized that the BST can be reliably used to produce a reference point correlating with hindfoot alignment.

## MATERIALS AND METHODS

This retrospective study, conducted in accordance with the Declaration of Helsinki and the Guidelines for Good Clinical Practice, included patients evaluated between January 2022 and October 2022. A total of 40 ankles from 40 patients with symptomatic osteoarthritis (OA) were assessed in our outpatient clinic. WBCT scans were performed as part of the preoperative evaluation.

Inclusion criteria were patients over 18 years old with advanced symptomatic OA, caused by post‐traumatic changes, ankle instability, or progressive collapsing foot disorder. Exclusion criteria included patients with metallic internal fixation devices that could produce metal artefacts or affect image quality. Patient images were retrieved from the medical centre's Picture Archiving and Communication System database, while demographics and relevant surgical and orthopaedic history were obtained from electronic medical records.

### WBCT Scan Protocol

A WBCT scan was performed as delineated by Efrima et al. [8], executed in a unipedal single stance to ensure consistency across all patients' scans. The protocol included a 0.2‐mm slice thickness, a 1‐mm slice increment and an effective radiation dose of 0.014 mSv per scan, utilizing the Planmed Verity system (Planmed Oy). Image analysis was conducted semi‐automatically using DISIOR Bonelogic Ortho Foot and Ankle Software (version 2.0), where a 3D model was reconstructed from WBCT‐generated DICOM files. An orthopaedic surgeon meticulously marked each bone at designated points, allowing the software to calculate axes and angles, including the hindfoot alignment angle, alpha angle, tibiotalar surface angle (TSA), beta angle and talar tilt (TT). The tibiotalar ratio (TTR) was calculated manually. Thus, it provides a comprehensive analysis of both intra‐articular and extra‐articular alignment.

### DM analysis

**Figure 1:**
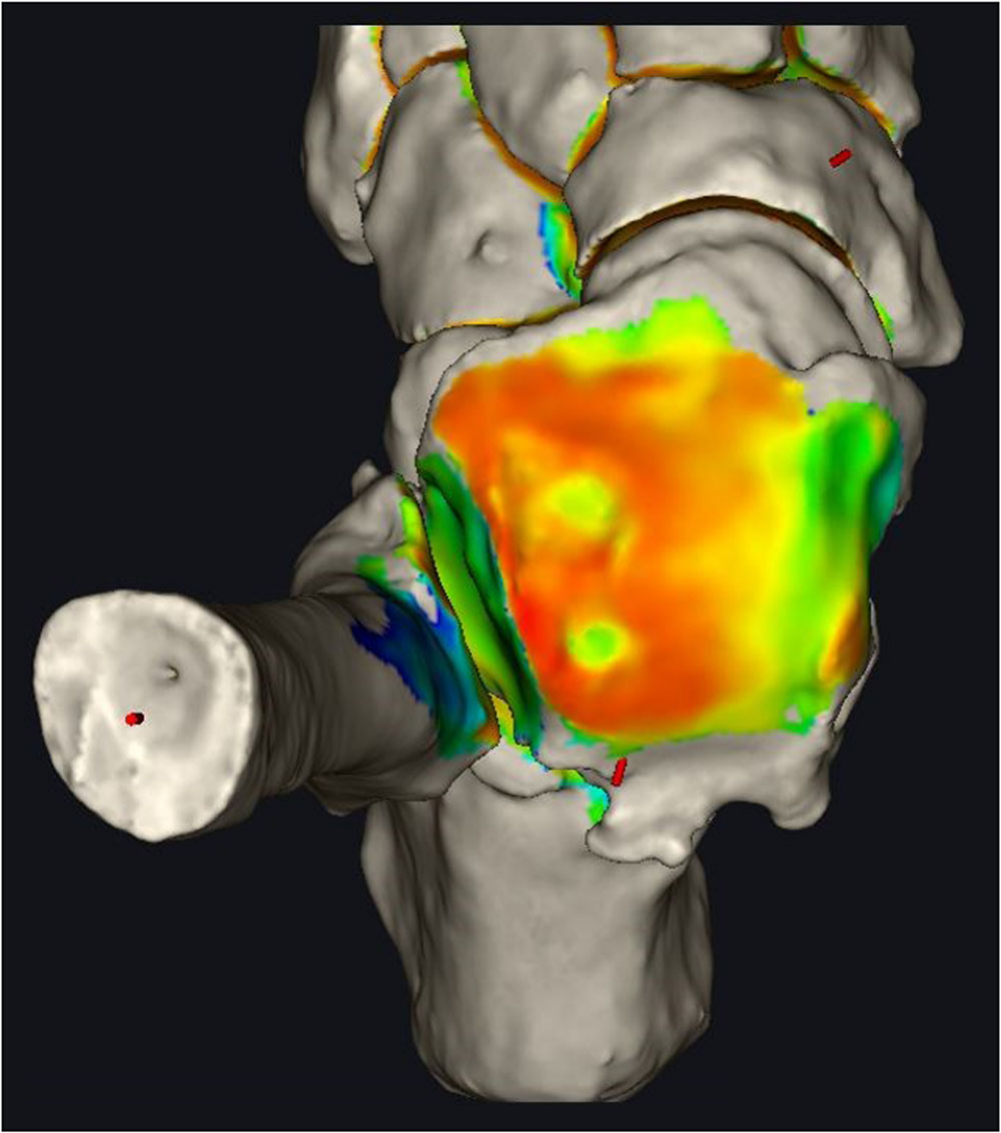
Example of distance mapping (DM) of the talar dome in osteoarthritis (OA) ankle. Warm colours represent closer bones in the joint (orange and red), and cold colours represent the longer distance between the tibia and talus (green and yellow). The two holes in the lateral dome are cysts.

The DM technique was employed to characterize and visualize the distance distribution between the articulating surfaces of the tibiotalar joint (Figure 1) [9]. Utilizing the updated DISIOR Bonelogic Ortho Foot and Ankle Software (version 2.1.1), the algorithm computed the relative distances between articular surfaces, assigning a colour spectrum to depict these distances visually. Thus, a colour pattern that resembles a topographic map, with red indicating minimal distances and blue denoting maximal distances, is created. A DM for the tibia‐talar surfaces was generated for each patient, with auto‐segmentations analyzed twice to ensure distance map consistency.

### Image processing

**Figure 2:**
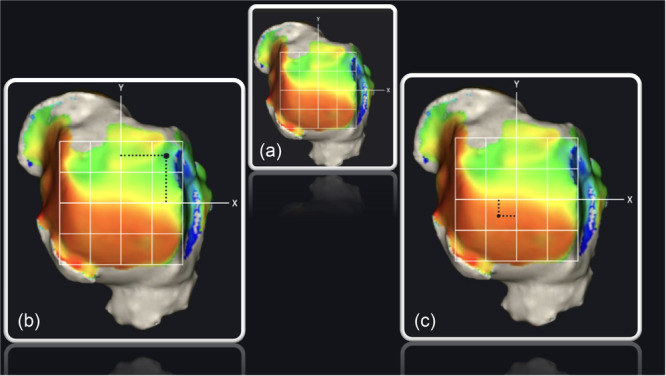
(a) Axial view of the 16‐square grid of a right foot. (b) Distance measurement of the individual weighted sum of the upper lateral square. (c) Total Weighted Sum of the talar dome.

A 3D ankle model and a semi‐automated report of angles and measurements were compiled for each patient. The study focused on the DM of the talar dome surface in axial view. The anatomical centre of the talar dome was determined by measuring the articular surface from posterior to anterior and from medial to lateral, with the intersection of these measurements deemed the anatomical centre. A rectangle was drawn to delineate the DM borders, and two lines were drawn to create *X‐* and *Y*‐axes, dissecting the talar dome into four primary quadrants: anterolateral (AL), anteromedial (AM), posterolateral (PL) and posteromedial (PM). Subsequently, each quadrant was divided into four equal parts, resulting in a grid of 16 identical quadrants grid, as illustrated in Figure 2a.

### DM pattern analysis—The BST

The BST was utilized to analyze the DM pattern of the talar dome using a 16‐square grid, intentionally excluding extra‐articular osteophytes from the analysis. For each square, the average intraarticular distance was semi‐automatically calculated by the DM algorithm and recorded in millimetres. Subsequently, a distance mapping coefficient (DMC) was determined for each square by subtracting the square's average intraarticular distance from one. Intraarticular cysts received a DMC equal to one, representing the highest possible DMC value. Furthermore, the relative position of each square's centre to the talar dome's central axis was determined, providing coordinates relative to both the *X*‐ and *Y*‐axes. Multiplying the DMC of each square by its relative coordinates to the central axis enabled the calculation of the individual weighted sum for each square along the *X*‐axis (WSQx) and *Y*‐axis (WSQy) (Figure 2b). Summing all WSQx and WSQy values and dividing by the total number of squares yielded two measurements: the total weighted sum X (TWSx) and total weighted sum Y (TWSy). These metrics represent the coordinates of the cumulative weighted sum in relation to the *X*‐ and *Y*‐axes, effectively mapping the weighted distribution's focal point on the articulating surface of the talar dome (Figure 2c).

### Data analysis

Two fellowship‐trained orthopaedic surgeons specializing in foot and ankle surgery calculated the TWSx and TWSy for each patient, producing a coordinate along the *X*‐ and *Y*‐axes for individual analysis. They were then instructed to classify the patients into four groups—varus, valgus, anterior and posterior—based on predefined criteria. The patient's placement into the varus group was determined if their TWSx was located within the AM or PM quadrants. Conversely, placement into the valgus group required the TWSx to be within the AL or PL quadrants. For sagittal categorization, a patient was classified into the anterior group if the TWSy fell within the AM and AL quadrants or into the posterior group if found within the PL and PM quadrants. Patients whose TWSx or TWSy equalled 0 were assigned to the neutral group. After a minimum of 2 months from the first measurement, a second measurement took place. Consequently, interobserver and intraobserver agreement were measured.

After classification, the influence and correlation of the coronal alignment on the TWSx and the sagittal alignment on the TWSy in the foot and ankle were evaluated. This evaluation used the semiautomatic measured angles—*α* angle, TSA, HFA, tibiotalar tilt and the talocalcaneal angle for coronal alignment assessment, and the *β* angle and TTR for sagittal alignment assessment. Thus, it provides a comprehensive assessment of each patient's foot and ankle alignment in correlation with their DM patterns.

### Statistical analysis

Continuous data are presented by means and standard deviation (SD) for normally distributed data and in median and interquartile range for non‐normally distributed data. The normal distribution of the data was tested using the Shapiro–Wilk test. For each rater, intra‐rater reliability between the two readings and inter‐rater reliability between the two raters were calculated using interclass correlation coefficient (ICC) and presented with a 95% confidence interval. ICC was calculated for a single measure using a two‐way random effect model of absolute agreement. ICC < 0.5 was interpreted as poor agreement, ICC 0.5–0.75 was interpreted as moderate agreement, ICC 0.75–0.9 was considered good agreement and ICC 0.9–1 was interpreted as excellent agreement. The independent sample *t* test was used to compare normally distributed data, and the Mann–Whitney *U* test was used to compare non‐normally distributed data. *p* Value < 0.05 was considered significant. The correlation was tested using Pearson and Spearman for normally and non‐normally distributed data, respectively. For correlation comparisons, a *p* value < 0.001 was considered significant.

The sample size was calculated a priori, drawing on the methodology outlined in the study by Królikowska et al. This calculation considered the requirement for three measurements per participant, aiming for a statistical power of 90% and a significance level (*α*) of 0.05, while targeting a minimum ICC of 0.50. Based on these parameters, it was determined that a minimum of 15 participants would be necessary for the analysis. An additional 30% of participants were included in the calculation to account for potential dropouts or missing data. Consequently, a diverse group of 20 participants is deemed adequate for the study. Statistical analysis was performed using SPSS statistical software IBM SPSS Statistics software (IBM Corp. Released 2013. IBM SPSS Statistics, Version 29.0. Armonk, NY: IBM Corp.).

## RESULTS

**Graph 1:**
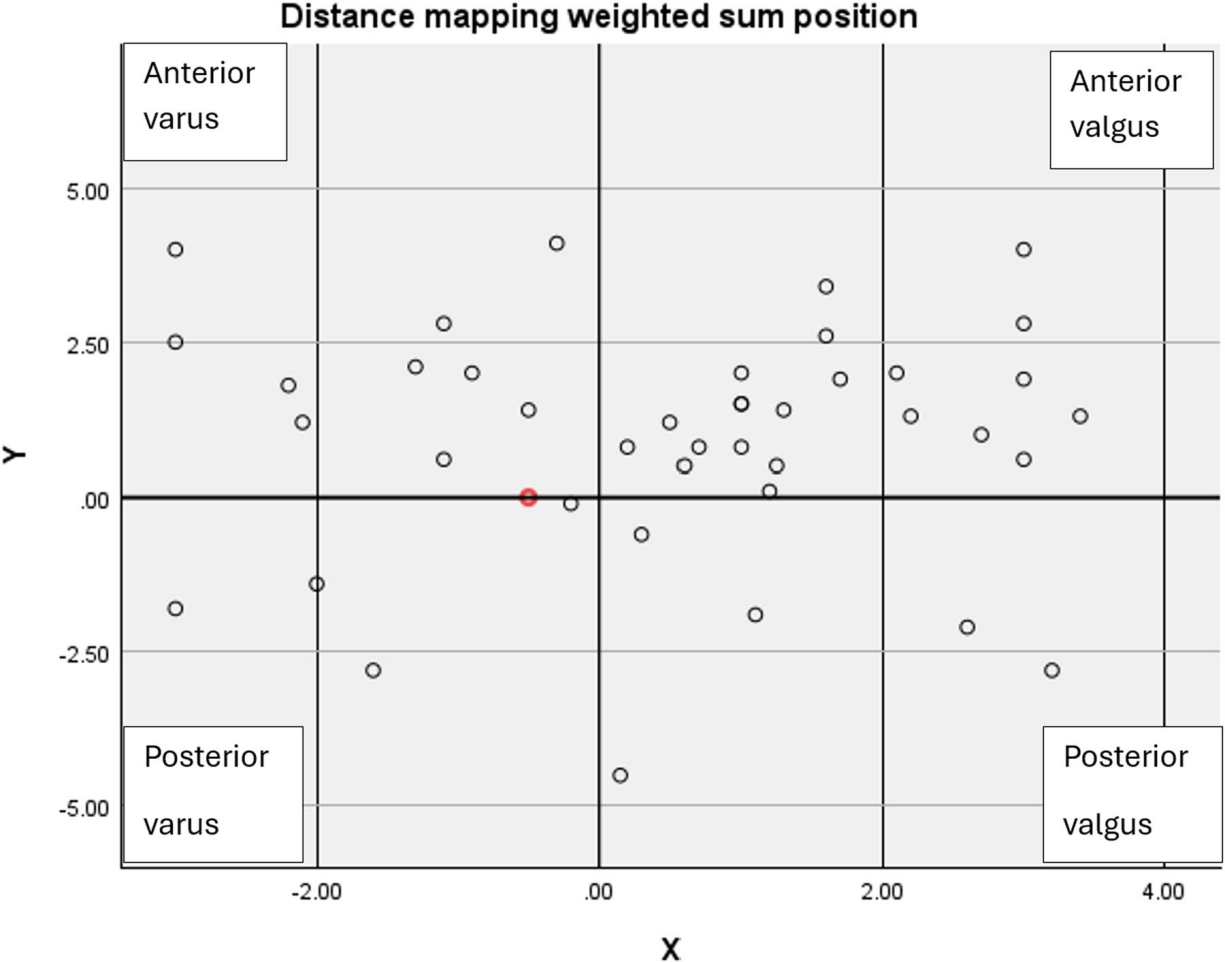
Demonstrates the distance mapping weighted sum points with respect to the *x* and *y*‐axes.

Patient demographic and clinical characteristics are detailed in Table 1. Twenty‐five patients were included in the valgus group, and 15 patients were in the varus group. The anterior group included 31 patients, and the posterior group included 8 patients. One patient was in a neutral position with respect to the *y*‐axis. Twenty patients in the valgus group were included in the anterior group and five in the posterior group. Eleven patients in the varus group were included in the anterior group and three patients in the posterior group (Graph 1, 3). The overall measurements and each group's individual angle measurements are summarized in Table 2.

**Table 1:** Patient demographics and clinical characteristics.

Characteristic	Value
Total patients	40
Males	27
Females	13
Mean age	58 ± 13 years
Aetiology	–
Posttraumatic	23
Instability	10
Progressive collapsing foot deformity	7
Osteoarthritis grade according to Richter et al.	–
Richter Grade 1	7
Richter Grade 2	26
Richter Grade 3	4
Richter Grade 4	3

**Table 2:** Summary of statistical findings in ankle osteoarthritis analysis using the Battleship technique.

Parameter	Overall	Valgus group	Varus group	Anterior group	Posterior group	*p* value
*α* angle (degrees)	90 [IQR 82–98]	89 [IQR 81–97]	91 [IQR 83–99]	N/A	N/A	*p* = 0.047
TSA angle (degrees)	88 [IQR 77‐99]	94 [IQR 84–104]	87 [IQR 75–99]	N/A	N/A	*p* < 0.001
HFA (degrees)	20 [IQR 0–20]	21 [IQR 4–38]	9 [IQR 11–29]	N/A	N/A	*p* = 0.003
Talocalcaneal angle (degrees)	19 ± 2	20 ± 14	18 ± 14	N/A	N/A	N/A
Talar tilt (degrees)	N/A	4 ± 6	−2 ± 5	N/A	N/A	*p* = 0.003
*β* angle (degrees)	85 ± 7	N/A	N/A	84 ± 6	93 ± 2	*p* < 0.001
TTR (mm)	43 ± 7	N/A	N/A	42 ± 6	54 ± 6	*p* < 0.001

Abbreviations: HFA, hindfoot alignment angle; IQR, interquartile range; TSA, tibiotalar surface angle.

The mean intra‐rater agreement for the first rater was 0.9 (*p* < 0.001), and the intra‐rater agreement for the second rater was 0.9 (*p* < 0.001). The inter‐rater agreement between the raters in the first reading was 0.9 (*p* < 0.001). Finally, the inter‐rater agreement between the raters in the second reading was 0.9 (*p* < 0.001) (Table 3).

**Table 3:** Intra‐rater and inter‐rater agreement.

Measurement	Rater	Agreement coefficient	*p* value
Intra‐rater agreement	First rater	0.923	<0.001
Intra‐rater agreement	Second rater	0.912	<0.001
Inter‐rater agreement	First reading	0.903	<0.001
Inter‐rater agreement	Second reading	0.917	<0.001

When comparing the three‐coronal angle between the coronal groups, a statistically significant difference was found in all four angles, including *α* angle (*p* = 0.047), TSA (*p* < 0.001), TT (*p* = 0.003) and HFA (*p* = 0.041). There was a statistically significant difference between the two sagittal groups in TSA angle (*p* < 0.001) and *β* angle (*p* < 0.001). Comparing the two sagittal angles to the sagittal groups yielded a statistically significant difference between the posterior and anterior groups on both *β* and TTR measurements (*p* < 0.001) (Table 2).

A significant correlation was found between the distance TWSx to TSA (*r* = 0.6) (*p* < 0.001) and TT (*r* = −0.6) (*p* < 0.001). A significant correlation was also found between TWSy to *β* angle (*r* = 0.2) (*p* < 0.001) and TTR (*r* = −0.4) (*p* < 0.001) (Table 4).

**Table 4:** Correlations between distance mapping weighted sum location and ankle alignment parameters.

Correlation between	Coefficient (*r*)	*p* value
TWSx and talar surface angle	0.628	<0.001
TWSx and talar tilt angle	−0.571	<0.001
TWSy and *β* angle	0.2	<0.001
TWSy and tibiotalar ratio	−0.385	<0.001

Abbreviations: TWSx, total weighted sum X; TWSy, total weighted sum Y.

## DISCUSSION

This study employed the BST to calculate the DM‐weighted sum. The most important finding is excellent interobserver reliability, suggesting that the BTS is a dependable method for identifying the DM‐weighted sum. Furthermore, the study uncovered significant differences in TSA, TT, *α* angle and HFA among patients with a TWSx located in the varus quadrants to those in the valgus quadrants. Similarly, patients with a TWSy in the anterior quadrants exhibited statistically significant differences in TTR and β Angle compared to those in the posterior quadrants. Additionally, the study identified a moderate to strong correlation between TWSx and both TSA and TT.

The introduction of WBCT image analysis software and distance mapping has significantly increased the use of complex 3D models for clinical evaluations, preoperative planning and post‐operative follow‐up [1, 4, 8, 10, 13, 14, 17]. However, despite these advancements, the surgical community largely continues to use 2D anatomical axes of bones as the primary reference points [6, 10]. This study reveals that the BST provides a reliable and reproducible method to determine the DM‐weighted sum. More importantly, it establishes a link between the weighted sum and foot and ankle alignment, suggesting that this measure could be an additional reference for evaluating and correcting complex intraarticular deformities. This finding points to the possibility of leveraging new technological advancements alongside conventional clinical approaches to improve the assessment and management of foot and ankle conditions. However, the practical application of these insights in routine clinical settings remains to be fully explored.

**Figure 3:**
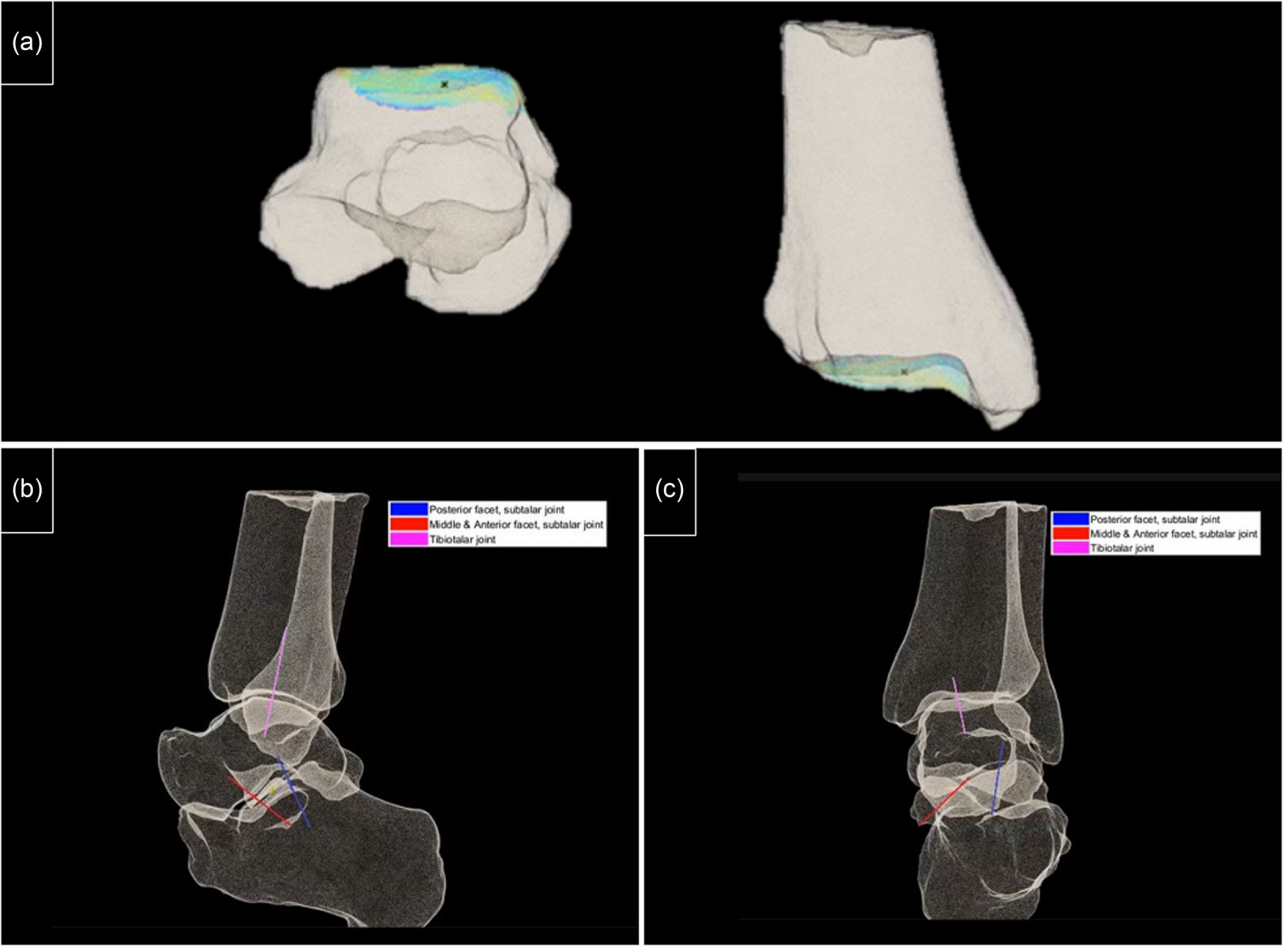
A potential use of the Battleship technique. (a) Location of the distance map weighted sum across the two sides of the tibio‐talar joint. (b) Tibiotalar joint's distance map axis and the subtalar posterior, medial, and anterior facets in sagittal view. (c) Distance map axis of the tibiotalar joint and the subtalar posterior, medial, and anterior facets in coronal views.

This study reconfirms a significant correlation between foot and ankle alignment and DM patterns [16]. By employing the BST, we calculated the DM‐weighted sum, accurately locating it along the *X*‐ and *Y*‐axes. Although our focus was on assessing the weighted sum of the talar dome, the potential applications of this method extend further. Theoretically, it could be applied to evaluate DM patterns across various joints, enabling the calculation of weighted sums on both articulating surfaces (Figure 1, 3). This possibility leads to the theoretical creation of an axis that connects the weighted sum points across a joint. Such an axis, effectively a *Z*‐axis, would link the *X* and *Y* reference points on either side of the joint, delineating the DM axis (Figure 1, 3). This newly proposed axis could serve as a crucial reference for preoperative planning in complex foot and ankle realignment surgeries. By integrating this approach, we can deepen our understanding of joint alignment and DM patterns, paving the way for more targeted and effective surgical interventions.

Corazza et al. [3] employed DM to analyze the variations in intraarticular distance within the joint space during passive ankle flexion, drawing parallels between their findings and intraarticular pressure maps. Similarly, Peiffer et al. utilized WBCT and FEA to establish a link between alignment, contact area, and pressure maps. The potential of DM as a straightforward and non‐invasive method for assessing intraarticular pressure has captured the interest of many researchers. While the primary focus of this study does not encompass the direct assessment of intraarticular joint pressure, it is noteworthy that many investigations rely on the centre of pressure (COP) equation to explore joint pressure dynamics [19]. The COP methodology, which shares conceptual similarities with the BST, involves segmenting the joint surface into smaller sections. The pressure attributed to each section is calculated by multiplying the unit's pressure by its distance from the joint's centre. Given these parallels, it is imperative to conduct further research comparing the DM‐weighted sum with COP values. Establishing a correlation between these two reference points could have profound implications, potentially revolutionizing our understanding and calculating joint pressure vectors across the joint. the preoperative plan could be designed not only to balance the alignment but also to balance the pressure vectors across the joint line.

This study is subject to several limitations that warrant consideration. First, image acquisition was performed in a unipedal stance, which may accentuate any existing deformities. Additionally, the study involved a relatively small cohort (*n* = 40), suggesting that findings should be validated through larger‐scale research. Moreover, patients who had undergone internal fixation were excluded to mitigate the influence of metal artefacts, potentially introducing a selection bias. Future studies should include post‐surgical populations to ensure a more comprehensive understanding of the findings.

## CONCLUSION

This study demonstrated the efficacy of the BST in calculating the DMWS, highlighting its reliability through excellent interobserver agreement. Significant correlations were found between the DMWS and variations in foot and ankle alignment, suggesting its potential as a novel reference point for assessing complex deformities. Clinically, the BST provides a standardized, non‐invasive method to evaluate intraarticular contact patterns, offering critical insights for preoperative and post‐operative assessment. Its integration into routine practice could improve the precision of surgical interventions, particularly in complex realignment procedures, and improve long‐term functional outcomes for patients with foot and ankle pathologies.

## AUTHOR CONTRIBUTIONS

All listed authors have contributed substantially to this work. Ben Efrima performed the study conception and design, literature review, data collection, data analysis and statistical analysis, manuscript preparation and manuscript review. Agustin Barbero performed the study conception and design, data collection, figures and manuscript review. Amit Benady assisted with the manuscript preparation. Yair Green Halimi assisted with the manuscript preparation. Jari Dahmen assisted with the manuscript preparation. Gino M. M. J. Kerkhoffs assisted with the manuscript preparation. Jon Karlsson assisted with the manuscript preparation. Camila Maccario assisted with the data collection. Cristian Indino assisted with the data collection. Federico G. Usuelli assisted with the manuscript preparation and data collection. All authors read and approved the final manuscript.

## CONFLICT OF INTEREST STATEMENT

Federico G. Usuelli reports relationships with Zimmer Biomet, Arthrex Inc., Episurf, Planmed Oy, Geistlich Pharma AG and BRM Trust for consulting/advisory roles and speaking/lecture fees; with Paragon 28 Inc. for consulting/advisory roles, employment, paid expert testimony and speaking/lecture fees; and holds a membership as International Editor for Foot and Ankle International. The remaining authors declare no conflicts of interest.

## ETHICS STATEMENT

Ethical approval for this study was obtained in accordance with the Declaration of Helsinki and the Guidelines for Good Clinical Practice (EndSP(13/INT/2016)). This study was performed at Humanitas San Pio X Hospital, Milan, Italy. Patient consent was waived for this retrospective study as all data were anonymized, and no identifying information was used, in accordance with institutional board guidelines.

## Data Availability

The data supporting this study are available upon reasonable request from the first author. Access is restricted to protect patient privacy.
